# Elective surgical services need to start planning for summer pressures

**DOI:** 10.1093/bjs/znad033

**Published:** 2023-03-23

**Authors:** GreenSurg Collaborative, Maria Picciochi, James C Glasbey, Elizabeth Li, Sivesh K Kamarajah, Dmitri Nepogodiev, Joana F F Simoes, Aneel Bhangu, Arjun Nathan, Nizar S M Ismail, Amer J Durrani, Fanourios Georgiades, Ignatius Liew, Mamun D Dornseifer, Chetan D Parmar, Angelos G Kolias, Efstratia A Baili, Ashwani Kumar Nugur, Erminia Albanese, Marios Ghobrial, Andreas K Demetriades, Joseph P Attwood, Baljit Singh, Ciaran M Barlow, Sheila M Fraser, Manas K Dube, Avinash Aujayeb, Dinesh K Thekkinkattil, Abraham J Botha, Tosin O Akinyemi, W K E Peng, Salah A Hammouche, Muyed K A Mohamed, Mahmoud K A Elmesalmi, Maria G Cannoletta, Kai Yuen Wong, Hassan M T Fawi, Suk F Cheng, Funlayo O Odejinmi, Hugo R M Layard Horsfall, Nikolaos Machairas, Rory C Cuthbert, Shahbaz S Malik, Rory G Callan, Richard J Egan, Nader N Moawad, David W Ferguson, Nathan Grundy, Michelle L Collins, Jonathan B T Herron, Chetan Khatri, Sophia E Lewis, Tariq Alhammali, Andrew J Beamish, Kiran K Singisetti, Joseph Shalhoub, Chung S Chean, Rajesh Sivaprakasam, Sreekar Devarakonda, Miles W Benjamin, Sivesh K Kamarajah, James Ashcroft, Pierfrancesco Lapolla, Christin Henein, Baljit Singh, Cillian T Forde, Mohammad Zain Sohail, Rachael J Clegg, Zoe M Seymour, Stavros V Parasyris, Nikolaos Dimitrokallis, Benjamin J Davies, Waleed F A Fahmy, Obafemi K Wuraola, Athanasios Serlis, Binay Gurung, Andrew J Kelly, Rishi Talwar, Paul S Cullis, Dale J Gracie, Markus P Baker, George W V Cross, Wai Wai Win Mar, Raashad Hasan, Dimitri J Pournaras, Cho Ee Ng, Ashok R Ramasamy, Amir N A Iskandar, James C Glasbey, Haaris A Shiwani, Sujesh Bansal, Stephen F McAleer, Omar Ahmed, Nader N Moawad, Umakanth R Kempanna, John-Joe Reilly, Richard J Davies, Sibtain Anwar, Grant A Harris, Usama Ahmed, Kareem T Elsanhoury, Wen Jie Chin, Nikhil Kumar Ponugoti, Javaria Faiz, Amer J Durrani, Mohit Bhatia, Jonathon R C Sheen, Imran H Yusuf, Ziyan Sheng, Grant D Stewart, Shafquat Zaman, Aloka S Danwaththa Liyanage, Karthikeyan P Iyengar, Ravi Aggarwal, Setthasorn Z Y Ooi, Ayesha Mahmud, Mingzheng Aaron Goh, James M D Wheeler, Nicola J Eardley, Michael El Boghdady, Delvene Soares, Alexander D O'Connor, Ankur D Kariya, Filip Fryderyk Brzeszczyński, Joshua L Moreau, Abdel Saed, Isobel Pilkington, Devaraj M Navaratnam, Neil A Ryan, Hooman Soleymani Majd, Lamiese Ismail, Hemina B Shah, Akib M Khan, Paul C Nankivell, Waleed Fayez Ali Fahmy, Robert W Tyler, Leandro Siragusa, Syed S Mannan, Giorgio Bogani, Jibran Abbasy, Piergiorgio Solli, Nadine Di Donato, Josh R Burke, Abdul Hakeem, Firas Aljanadi, Alexander J Baldwin, Mohamed Bekheit, Peter P Bobak, Matyas Fehervari, Fabio Barra, Mohamed A Thaha, Nadir Syed, James B Olivier, Khaled A K Mohammed, Kate J Williams, Tatiana Martin, Aman S Coonar, Michael W S Ho, Mark W Yao, Alexandros Konstantinos Charalabopoulos, Porfyrios G Korompelis, Kay Anne Mak, Abdelrahman A A Elsayed, Eve R Hawley, Ahmed Y Azzam, Alan J B Kirk, Ahmed E Sherif, Mostafa K A Hussein, James A Blair, Yirupaiahgari K S Viswanath, Simon J Cole, Dheeraj S Attarde, Anna Y Allan, Ioannis N Gerogiannis, Shiva Dindyal, Muhammad H Siddique, Saidah Sahid, Jonathan J Neville, David N Naumann, Matthew H V Byrne, Sean M A Garcia, Ali Yasen Y Mohamedahmed, Alan A Askari, Joerg M Pollok, Hani J Marcus, Kapil Sahnan, Mohamed A Thaha, Qamar Mustafa, Ruben P Thumbadoo, Angelos G Kolias, Ketan Agarwal, Sean Khedar Ramcharan, Mehran Lashari, Mostafa E A Abdelkarim, Toby M Noton, Bilal H Kirmani, Robert D J Whitham, Sofia Anastasiadou, Rute S S Castelhano, Sanad Saad, Gakul Dr Bhatta, Chetan D Parmar, Antonio Leyte Golpe, Rucira Ooi, Emily C M McKenzie, Kenneth N Linton, Khalid M Bhatti, Shyama S Chadha, Liam N Phelan, Alvaro Bedoya Ronga, Vladislav Kutuzov, Mohammed Jibreel Mohammed, Sharan H Sambhwani, Catrin Sohrabi, Raghavan Vidya, Jaskiran K Gill, Lisa S Rampersad, Bincy Merin Zacharia, Waheeb A K Al-Azzani, Omar Pathmanaban N Pathmanaban, Rachel Sarah Olive, Fahad S Hossain, Jessica Harvey, Naren K Kumaran, Annamaria Minicozzi, Andrew Neil Wheelton, Victoria A Evans, Andrew D Beggs, Omar M Ismail, Chandra Shekhar Biyani, Shaikh S Seraj, Mohammed Deputy, Eltayeb B E Shammeseldin, Wafi Mohammed W M Mohammed, Mohamed Onsa, Yizhe Lim, Ahmad Riyadh Abdulsaheb Al-Shaye, Mujahid Gasemelseed Fadlallah, Hash Al-Musawi, Umar B J Yousuf, Safia Zahir Ahmed, Alexandros Laios, Aliabbas Moosa, Zoe Li, Peter J Hutchinson, Abdalla Hassan Abdalla Hassan, Shreya M Kulkarni, Shihab A Chowdhury, Ahmed Y Ammar, Tarig Hassan Ahmed, Raimundas A Lunevicius, Dimitrios Angelou, Edward J Caruana, Panna K Patel, Stephen J Bromage, Panagiotis Kapsampelis, Khaled M Sarraf, Antonios Nicolaos Athanasiou, Jai Relwani, James E Tomlinson, Amarkumar D Rajgor, Pedram Panahi, Rachael V Collins

*Dear Editor*

The COVID-19 pandemic exposed the fragility of elective surgical and anaesthesia services, resulting in millions of operations being cancelled across the world^1^. As health systems plan post-COVID recovery of elective surgical services, they should identify potential future external risks to surgical services^2^. Extreme weather events resulting from climate change could present an increasing challenge to healthcare systems^3^; surges in injuries and cardiorespiratory complications during heatwaves could reduce capacity to deliver elective care^4^. The aim of this study was to determine the impact of summer pressures on the delivery of elective surgery in the UK.

The authors conducted a cross-sectional survey of surgeons, anaesthetists, and critical care doctors who worked during the UK heatwave of 16–19 July 2022. A total of 271 responses were received from across 20 specialties in 140 UK hospitals. One in five respondents (50 of 271, 18.5 per cent) reported that the heatwave directly resulted in the cancellation of elective surgery. A further third (96 of 271, 35.1 per cent) anticipated that cancellations were likely in the event of a prolonged heatwave. Factors contributing to heatwave-related cancellations included staff shortages (reported by 35.8 per cent of 271 respondents), unsafe theatre environments (30.3 per cent), and bed shortages (22.1 per cent) (*Fig. 1*).

**Fig. 1:**
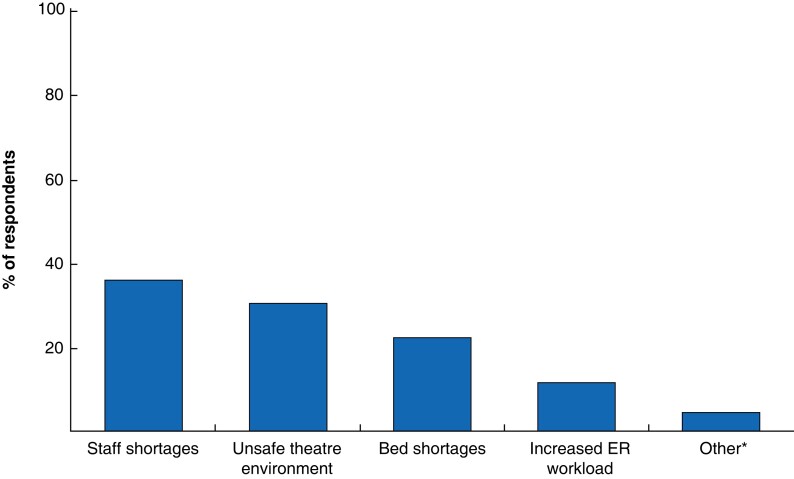
Factors contributing to heatwave-related cancellations of elective surgery *IT problems caused by the heat, patients unwilling to have surgery owing the heatwave. ER, emergency room.

Surgical services were poorly prepared for heatwaves. Ambient temperature could not be controlled in 41.0 per cent of operating theatres. Most hospitals (119 of 140, 85.0 per cent) lacked summer pressure plans to maintain elective surgical safety and capacity. Some 96 respondents (35.4 per cent) reported making adaptations to maintain the routine surgical activity during the heatwave (*Table S1*).

These data demonstrate that even short heatwaves may result in widespread disruption to surgical services. As hospitals tackle post-COVID surgery backlogs^5^, they must consider how to safeguard against further climate change-related disruption to the delivery of surgical services. This should be included in the preparation of summer pressure plans to improve the resilience of elective surgery services.

## Collaborators

GreenSurg Collaborative: Maria Picciochi, James C Glasbey, Elizabeth Li, Sivesh K Kamarajah, Dmitri Nepogodiev, Joana FF Simoes, Aneel Bhangu, Arjun Nathan, Nizar S M Ismail, Amer J Durrani, Fanourios Georgiades, Ignatius Liew, Mamun D Dornseifer, Chetan D Parmar, Angelos G Kolias, Efstratia A Baili, Ashwani Kumar Nugur, Erminia Albanese, Marios Ghobrial, Andreas K Demetriades, Joseph P Attwood, Baljit Singh, Ciaran M Barlow, Sheila M Fraser, Manas K Dube, Avinash Aujayeb, Dinesh K Thekkinkattil, Abraham J Botha, Tosin O Akinyemi, W K E Peng, Salah A Hammouche, Muyed K A Mohamed, Mahmoud K A Elmesalmi, Maria G Cannoletta, Kai Yuen Wong, Hassan MT Fawi, Suk F Cheng, Funlayo O Odejinmi, Hugo RM Layard Horsfall, Nikolaos Machairas, Rory C Cuthbert, Shahbaz S Malik, Rory G Callan, Richard J Egan, Nader N Moawad, David W Ferguson, Nathan Grundy, Michelle L Collins, Jonathan BT Herron, Chetan Khatri, Sophia E Lewis, Tariq Alhammali, Andrew J Beamish, Kiran K Singisetti, Joseph Shalhoub, Chung S Chean, Rajesh Sivaprakasam, Sreekar Devarakonda, Miles W Benjamin, Sivesh K Kamarajah, James Ashcroft, Pierfrancesco Lapolla, Christin Henein, Baljit Singh, Cillian T Forde, Mohammad Zain Sohail, Rachael J Clegg, Zoe M Seymour, Stavros V Parasyris, Nikolaos Dimitrokallis, Benjamin J Davies, Waleed F A Fahmy, Obafemi K Wuraola, Athanasios Serlis, Binay Gurung, Andrew J Kelly, Rishi Talwar, Paul S Cullis, Dale J Gracie, Markus P Baker, George WV Cross, Wai Wai Win Mar, Raashad Hasan, Dimitri J Pournaras, Cho Ee Ng, Ashok R Ramasamy, Amir N A Iskandar, James C Glasbey, Haaris A Shiwani, Sujesh Bansal, Stephen F McAleer, Omar Ahmed, Nader N Moawad, Umakanth R Kempanna, John-Joe Reilly, Richard J Davies, Sibtain Anwar, Grant A Harris, Usama Ahmed, Kareem T Elsanhoury, Wen Jie Chin, Nikhil Kumar Ponugoti, Javaria Faiz, Amer J Durrani, Mohit Bhatia, Jonathon RC Sheen, Imran H Yusuf, Ziyan Sheng, Grant D Stewart, Shafquat Zaman, Aloka S Danwaththa Liyanage, Karthikeyan P Iyengar, Ravi Aggarwal, Setthasorn Z Y Ooi, Ayesha Mahmud, Mingzheng Aaron Goh, James M D Wheeler, Nicola J Eardley, Michael El Boghdady, Delvene Soares, Alexander D O'Connor, Ankur D Kariya, Filip Fryderyk Brzeszczyński, Joshua L Moreau, Abdel Saed, Isobel Pilkington, Devaraj M Navaratnam, Neil A Ryan, Hooman Soleymani Majd, Lamiese Ismail, Hemina B Shah, Akib M Khan, Paul C Nankivell, Waleed Fayez Ali Fahmy, Robert W Tyler, Leandro Siragusa, Syed S Mannan, Giorgio Bogani, Jibran Abbasy, Piergiorgio Solli, Nadine Di Donato, Josh R Burke, Abdul Hakeem, Firas Aljanadi, Alexander J Baldwin, Mohamed Bekheit, Peter P Bobak, Matyas Fehervari, Fabio Barra, Mohamed A Thaha, Nadir Syed, James B Olivier, Khaled A K Mohammed, Kate J Williams, Tatiana Martin, Aman S Coonar, Michael W S Ho, Mark W Yao, Alexandros Konstantinos Charalabopoulos, Porfyrios G Korompelis, Kay Anne Mak, Abdelrahman AA Elsayed, Eve R Hawley, Ahmed Y Azzam, Alan JB Kirk, Ahmed E Sherif, Mostafa K.A Hussein, James A Blair, Yirupaiahgari KS Viswanath, Simon J Cole, Dheeraj S Attarde, Anna Y Allan, Ioannis N Gerogiannis, Shiva Dindyal, Muhammad H Siddique, Saidah Sahid, Jonathan J Neville, David N Naumann, Matthew H V Byrne, Sean MA Garcia, Ali Yasen Y Mohamedahmed, Alan A Askari, Joerg M Pollok, Hani J Marcus, Kapil Sahnan, Mohamed A Thaha, Qamar Mustafa, Ruben P Thumbadoo, Angelos G Kolias, Ketan Agarwal, Sean Khedar Ramcharan, Mehran Lashari, Mostafa EA Abdelkarim, Toby M Noton, Bilal H Kirmani, Robert D J Whitham, Sofia Anastasiadou, Rute S S Castelhano, Sanad Saad, Gakul Dr Bhatta, Chetan D Parmar, Antonio Leyte Golpe, Rucira Ooi, Emily C M McKenzie, Kenneth N Linton, Khalid M Bhatti, Shyama S Chadha, Liam N Phelan, Alvaro Bedoya Ronga, Vladislav Kutuzov, Mohammed Jibreel Mohammed, Sharan H Sambhwani, Catrin Sohrabi, Raghavan Vidya, Jaskiran K Gill, Lisa S Rampersad, Bincy Merin Zacharia, Waheeb A K Al-Azzani, Omar Pathmanaban N Pathmanaban, Rachel Sarah Olive, Fahad S Hossain, Jessica Harvey, Naren K Kumaran, Annamaria Minicozzi, Andrew Neil Wheelton, Victoria A Evans, Andrew D Beggs, Omar M Ismail, Chandra Shekhar Biyani, Shaikh S Seraj, Mohammed Deputy, Eltayeb B E Shammeseldin, Wafi Mohammed W M Mohammed, Mohamed Onsa, Yizhe Lim, Ahmad Riyadh Abdulsaheb Al-Shaye, Mujahid Gasemelseed Fadlallah, Hash Al-Musawi, Umar B J Yousuf, Safia Zahir Ahmed, Alexandros Laios, Aliabbas Moosa, Zoe Li, Peter J Hutchinson, Abdalla Hassan Abdalla Hassan, Shreya M Kulkarni, Shihab A Chowdhury, Ahmed Y Ammar, Tarig Hassan Ahmed, Raimundas A Lunevicius, Dimitrios Angelou, Edward J Caruana, Panna K Patel, Stephen J Bromage, Panagiotis Kapsampelis, Khaled M Sarraf, Antonios Nicolaos Athanasiou, Jai Relwani, James E Tomlinson, Amarkumar D Rajgor, Pedram Panahi, Rachael V Collins.

## Supplementary Material

znad033_Supplementary_Data

## Data Availability

Anonymised data available upon request of the writing group, and successful completion of a Data Sharing Agreement through an Application Programming Interface (API) linked to the REDCap data server hosted at Birmingham Clinical Trials Unit at the University of Birmingham.
