# Supplementary material for: Elective surgical services need to start planning for summer pressures
Source: Br J Surg. 2023 Mar 23;110(4):508–10. doi: 10.1093/bjs/znad033 (PMC10364522; doi:10.1093/bjs/znad033)
Supplement: znad033_Supplementary_Data [file znad033_supplementary_data.docx]

# Elective surgical services need to start planning for ‘summer pressures’

# *GreenSurg Collaborative*

**Correspondence:**

Mr James Glasbey MBBCh BSc MRCS, NIHR Doctoral Research Fellow in Global Surgery,

NIHR Global Health Research Unit on Global Surgery, University of Birmingham, Institute of

Translational Medicine, Heritage Building, Mindelsohn Way, Birmingham, United Kingdom, B15 2TH. Email: j.glasbey@bham.ac.uk

**Supplementary Materials - Index**

| **Supplementary Figures and Tables** |  |
| --- | --- |
| Table S1 – Strategies adopted during the heatwave | *pag. 2* |
| **Supplementary Appendixes** |  |
| Authorship | *pag. 3-4* |
| **References** | *pag. 5* |

**Supplementary Figures and Tables**

# Table S1 – Strategies adopted during the heatwave

| **Strategy** | **n (%)** |
| --- | --- |
| Delayed discharge of high-risk patients | 35 (36.5%) |
| Changes to surgical teams | 26 (27.1%) |
| Selected lower risk patients to have surgery | 23 (24.0%) |
| Restricted surgical activity to day-case | 22 (22.9%) |
| Other* | 23 (24.0%) |

This table describes the strategies adopted during the heatwave. In total, 96 of 271 respondents changed their normal practice during that week. *Other: longer staff breaks, administration of extra fluids to the patients admitted, starting surgeries earlier in the morning.

# Supplementary Appendixes

# Authorship (all PubMed-citable)

**Central group and Writing group** (*denotes joint first authors): Maria Picciochi*, James C Glasbey*, Elizabeth Li, Sivesh K Kamarajah, Dmitri Nepogodiev, Joana FF Simoes, Aneel Bhangu (Overall guarantor)

**Collaborators**:

Arjun Nathan, Nizar S M Ismail, Amer J Durrani, Fanourios Georgiades, Ignatius Liew, Mamun D Dornseifer, Chetan D Parmar, Angelos G Kolias, Efstratia A Baili, Ashwani Kumar Nugur, Erminia Albanese, Marios Ghobrial, Andreas K Demetriades, Joseph P Attwood, Baljit Singh, Ciaran M Barlow, Sheila M Fraser, Manas K Dube, Avinash Aujayeb, Dinesh K Thekkinkattil, Abraham J Botha, Tosin O Akinyemi, W K E Peng, Salah A Hammouche, Muyed K A Mohamed, Mahmoud K A Elmesalmi, Maria G Cannoletta, Kai Yuen Wong, Hassan MT Fawi, Suk F Cheng, Funlayo O Odejinmi, Hugo RM Layard Horsfall, Nikolaos Machairas, Rory C Cuthbert, Shahbaz S Malik, Rory G Callan, Richard J Egan, Nader N Moawad, David W Ferguson, Nathan Grundy, Michelle L Collins, Jonathan BT Herron, Chetan Khatri, Sophia E Lewis, Tariq Alhammali, Andrew J Beamish, Kiran K Singisetti, Joseph Shalhoub, Chung S Chean, Rajesh Sivaprakasam, Sreekar Devarakonda, Miles W Benjamin, Sivesh K Kamarajah, James Ashcroft, Pierfrancesco Lapolla, Christin Henein, Baljit Singh, Cillian T Forde, Mohammad Zain Sohail, Rachael J Clegg, Zoe M Seymour, Stavros V Parasyris, Nikolaos Dimitrokallis, Benjamin J Davies, Waleed F A Fahmy, Obafemi K Wuraola, Athanasios Serlis, Binay Gurung, Andrew J Kelly, Rishi Talwar, Paul S Cullis, Dale J Gracie, Markus P Baker, George WV Cross, Wai Wai Win Mar, Raashad Hasan, Dimitri J Pournaras, Cho Ee Ng, Ashok R Ramasamy, Amir N A Iskandar, James C Glasbey, Haaris A Shiwani, Sujesh Bansal, Stephen F McAleer, Omar Ahmed, Nader N Moawad, Umakanth R Kempanna, John-Joe Reilly, Richard J Davies, Sibtain Anwar, Grant A Harris, Usama Ahmed, Kareem T Elsanhoury, Wen Jie Chin, Nikhil Kumar Ponugoti, Javaria Faiz, Amer J Durrani, Mohit Bhatia, Jonathon RC Sheen, Imran H Yusuf, Ziyan Sheng, Grant D Stewart, Shafquat Zaman, Aloka S Danwaththa Liyanage, Karthikeyan P Iyengar, Ravi Aggarwal, Setthasorn Z Y Ooi, Ayesha Mahmud, Mingzheng Aaron Goh, James M D Wheeler, Nicola J Eardley, Michael El Boghdady, Delvene Soares, Alexander D O'Connor, Ankur D Kariya, Filip Fryderyk Brzeszczyński, Joshua L Moreau, Abdel Saed, Isobel Pilkington, Devaraj M Navaratnam, Neil A Ryan, Hooman Soleymani Majd, Lamiese Ismail, Hemina B Shah, Akib M Khan, Paul C Nankivell, Waleed Fayez Ali Fahmy, Robert W Tyler, Leandro Siragusa, Syed S Mannan, Giorgio Bogani, Jibran Abbasy, Piergiorgio Solli, Nadine Di Donato, Josh R Burke, Abdul Hakeem, Firas Aljanadi, Alexander J Baldwin, Mohamed Bekheit, Peter P Bobak, Matyas Fehervari, Fabio Barra, Mohamed A Thaha, Nadir Syed, James B Olivier, Khaled A K Mohammed, Kate J Williams, Tatiana Martin, Aman S Coonar, Michael W S Ho, Mark W Yao, Alexandros Konstantinos Charalabopoulos, Porfyrios G Korompelis, Kay Anne Mak, Abdelrahman AA Elsayed, Eve R Hawley, Ahmed Y Azzam, Alan JB Kirk, Ahmed E Sherif, Mostafa K.A Hussein, James A Blair, Yirupaiahgari KS Viswanath, Simon J Cole, Dheeraj S Attarde, Anna Y Allan, Ioannis N Gerogiannis, Shiva Dindyal, Muhammad H Siddique, Saidah Sahid, Jonathan J Neville, David N Naumann, Matthew H V Byrne, Sean MA Garcia, Ali Yasen Y Mohamedahmed, Alan A Askari, Joerg M Pollok, Hani J Marcus, Kapil Sahnan, Mohamed A Thaha, Qamar Mustafa, Ruben P Thumbadoo, Angelos G Kolias, Ketan Agarwal, Sean Khedar Ramcharan, Mehran Lashari, Mostafa EA Abdelkarim, Toby M Noton, Bilal H Kirmani, Robert D J Whitham, Sofia Anastasiadou, Rute S S Castelhano, Sanad Saad, Gakul Dr Bhatta, Chetan D Parmar, Antonio Leyte Golpe, Rucira X X Ooi, Emily C M McKenzie, Kenneth N Linton, Khalid M Bhatti, Shyama S Chadha, Liam N Phelan, Alvaro Bedoya Ronga, Vladislav Kutuzov, Mohammed Jibreel Mohammed, Sharan H Sambhwani, Catrin Sohrabi, Raghavan Vidya, Jaskiran K Gill, Lisa S Rampersad, Bincy Merin Zacharia, Waheeb A K Al-Azzani, Omar Pathmanaban N Pathmanaban, Rachel Sarah Olive, Fahad S Hossain, Jessica Harvey, Naren K Kumaran, Annamaria Minicozzi, Andrew Neil Wheelton, Victoria A Evans, Andrew D Beggs, Omar M Ismail, Chandra Shekhar Biyani, Shaikh S Seraj, Mohammed Deputy, Eltayeb B E Shammeseldin, Wafi Mohammed W M Mohammed, Mohamed Onsa, Yizhe Lim, Ahmad Riyadh Abdulsaheb Al-Shaye, Mujahid Gasemelseed Fadlallah, Hash Al-Musawi, Umar B J Yousuf, Safia Zahir Ahmed, Alexandros Laios, Aliabbas Moosa, Zoe Li, Peter J Hutchinson, Abdalla Hassan Abdalla Hassan, Shreya M Kulkarni, Shihab A Chowdhury, Ahmed Y Ammar, Tarig Hassan Ahmed, Raimundas A Lunevicius, Dimitrios Angelou, Edward J Caruana, Panna K Patel, Stephen J Bromage, Panagiotis Kapsampelis, Khaled M Sarraf, Antonios nicolaos Athanasiou, Jai Relwani, James E Tomlinson, Amarkumar D Rajgor, Pedram Panahi, Rachael V Collins.

**References**

1. COVIDSurg Collaborative. Projecting COVID-19 disruption to elective surgery. *The Lancet.* 2022;399(10321):233-234.
2. COVIDSurg Collaborative. Elective Cancer Surgery in COVID-19-Free Surgical Pathways During the SARS-CoV-2 Pandemic: An International, Multicenter, Comparative Cohort Study. *J Clin Oncol.* 2021;39(1):66-78.
3. Hannah D, Bartington S, Bonsu NO, et al. *Keeping 1.5ºC Alive.* 2022.
4. The Lancet. 2022 heatwaves: a failure to proactively manage the risks. *The Lancet.* 2022;400(10350).
5. Nepogodiev D, Acharya R, Chaudhry D, et al. Forecasting waiting lists for elective procedures and surgery in England: a modelling study. *medRXiv.* 2022.
